# Estimation and correction of instrument artefacts in dynamic impedance spectra

**DOI:** 10.1038/s41598-020-80468-x

**Published:** 2021-01-14

**Authors:** Collins Erinmwingbovo, Fabio La Mantia

**Affiliations:** Energiespeicher- und Energiewandlersysteme, Universität Bremen, Bibliothekstr. 1, 28359 Bremen, Germany

**Keywords:** Analytical chemistry, Electrochemistry, Physical chemistry

## Abstract

Dynamic impedance spectroscopy is one of the most powerful techniques in the qualitative and quantitative mechanistic studies of electrochemical systems, as it allows for time-resolved investigation and dissection of various physicochemical processes occurring at different time scales. However, due to high-frequency artefacts connected to the non-ideal behaviour of the instrumental setup, dynamic impedance spectra can lead to wrong interpretation and/or extraction of wrong kinetic parameters. These artefacts arise from the non-ideal behaviour of the voltage and current amplifier (I/E converters) and stray capacitance. In this paper, a method for the estimation and correction of high-frequency artefacts arising from non-ideal behaviour of instrumental setup will be discussed. Using resistors, $[\hbox {Fe(CN)}_6]^{3-/4-}$ redox couple and nickel hexacyanoferrate nanoparticles, the effect of high-frequency artefacts will be investigated and the extraction of the impedance of the system from the measured dynamic impedance is proposed. It is shown that the correction allows acquiring proper dynamic impedance spectra at frequencies higher than the bandwidth of the potentiostat, and simultaneously acquire high precision cyclic voltammetry.

## Introduction

Electrochemical impedance spectroscopy (EIS) is a highly sensitive technique used for the elucidation of reaction mechanisms and determination of kinetic parameters^1–3^. The acquisition of qualitative and quantitative mechanistic information relies on the fitting of high-quality impedance data, acquired over a broad range of frequencies and potentials using physicochemical models^2^. However, cell and instrument setups have been reported as major sources of artefacts in impedance spectra^4–6^. These artefacts can result in the wrong interpretation of the reaction mechanism or the extraction of wrong kinetic parameters. Several methods for identifying artefacts arising from cell setup and procedures for avoiding and/or correcting these artefacts have been reported in literature^4,5,7–13^.

Following the works of Fletcher^7^ and Sadkowski et al.^8^, Battistel et al. proposed solutions to circumvent the artefacts occurring in a three-electrode cell setup^4^. It comprises of using a three-electrode cell in a coaxial geometry and a capacitive bridge between the working electrode and reference electrode^4^. Tran et al. with the help of simulations explained the use of low impedance reference electrode fabricated by connecting a platinum wire to the reference electrode through a capacitor, which has been used in circumventing high-frequency artefacts^14–16^. Low impedance reference electrodes were used to circumvent high-frequency artefacts during the acquisition of dynamic impedance spectra of cathode materials for aqueous batteries such as nickel hexacyanoferrate thin films and $\hbox {LiMn}_{2}\hbox {O}_{4}$ films^17,18^.

A zero-gap cell combined with by-pass modified and sensing electrodes was proposed by Stojadinovic and co-workers to minimize distortions arising from artefacts in a four-electrode cell setup^10,19^. The use of a four-probe setup to measure high impedance values (up to 10 G$\Omega$) to circumvent artefacts arising from four-electrode cell setup has been demonstrated by Fafilek et al.^5^. Finite element method (FEM) simulations have also been used as a means of identifying limiting conditions for reliable impedance measurement in three-electrode cell setup for lithium-ion batteries^20,21^. To avoid artefacts arising from geometric and electrochemical asymmetries, Klink et al. suggested the use of modified Swagelok cells with precisely aligned electrodes and a coaxially located reference electrode^22^.

The aforementioned methods reduce and/or eliminate artefacts arising from cell setups, but they do not address artefacts arising from the instrument setup. The instrument setup used for the data acquisition in EIS contains parts (cables, voltage and current amplifiers), which deviates from ideal behaviour at high frequencies^4^. Manufacturers use calibration to reduce such artefacts at high frequencies^23^. In classic EIS, data acquisition parameters, among which the current amplifier, are automatically optimized for each frequency by the software, thereby reducing instrumental artefacts. This is not possible in dynamic impedance spectroscopy, in which the perturbation signal contains all the frequencies at once. Dynamic impedance spectroscopy has been reported as a better alternative for investigating the kinetics of unstable electrochemical systems and electrochemical systems under non-stationary conditions^17,18,24–37^. However, instrumental artefacts are particularly troublesome in dynamic impedance spectroscopy due to two contrasting requirements: on one side the acquisition of good dc data (cyclic voltammetry) requires the selection of a low-current amplifier; on the other side, to reach high frequencies a high-current amplifier with high bandwidth has to be chosen.

In this paper, we show how, by estimating the transimpedance response of the instrument, it is possible to correct the instrumental artefacts at high frequencies and increase the bandwidth of the current amplifier. In particular, we will illustrate as practical examples, the measurement of dynamic impedance spectra of redox couples in solution and potassium (de-)insertion in nickel hexacyanoferrate.

### Sources of instrumental artefacts

Instrument setups used for acquisition of impedance spectra such as potentiostats contain components (voltage and current amplifiers), which deviate from their ideal behaviour at high frequencies thereby introducing artefacts to the measured impedance. The current amplifier of the potentiostat measures the current as a voltage drop through a resistor $R_{m}$. Switchable resistors of different magnitude are used to cover different current ranges, thus allowing for high precision measurements^38^. The value of $R_{m}$ determines also the voltage per full current range of the current amplifier, which in the instrument used in this work is 1 V. As an example, this translates to $R_{m}$ equal to 1 k$\Omega$ when the current range of 1 mA is selected. The achievable gain of the current amplifier depends on the input impedance (measured impedance) and the transimpedance of the *I*/*E* converter. The bandwidth of the *I*/*E* converter depends on the gain such that the greater the gain (lower current range), the lower the bandwidth of the *I*/*E* converter. Measuring impedance at frequencies approaching or exceeding the bandwidth of the *I*/*E* converter could result in the introduction of artefacts arising from the instrumentation. Therefore, it is important to investigate the bandwidth of the *I*/*E* converter at different current ranges to properly choose it in connection to the perturbation signal.

The voltage signal applied to the cell at high-frequency by the control loop in the potentiostat may be shifted in phase due to its response bandwidth^39^. The use of larger inputs and monitoring of the feedback signal to the cell has been suggested for circumventing this problem^2,39^. Another source of artefacts in impedance measurements connected to both instrument and cell setups is the stray capacitance. Stray capacitances have been reported to occur between CE–RE, RE—ground, RE–WE and WE—ground^23^. Despite the calibration done by manufacturers to eliminate these stray capacitance, it has been reported that electrochemical cells affect the value of the stray capacitances^23^. Here, as a low-impedance RE is used in the experiments, it is possible to ignore the stray capacitances between CE–RE and RE—ground, as explained in literature^23^.

### Theoretical description of the potentiostat

**Figure 1:**
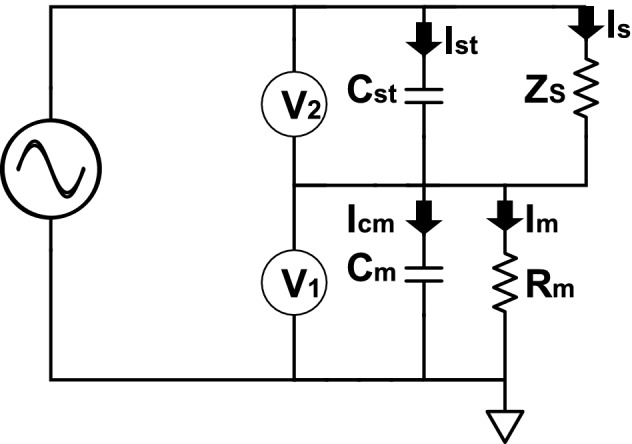
Simplified schematic diagram of a potentiostat in a two-electrode cell setup used for measuring the transimpedance and stray capacitance of the potentiostat used in this work. $Z_s$ denotes the impedance of the system being measured, $I_{s}$ is the current flowing through $Z_{s}$, $C_{st}$ is the stray capacitance and $I_{st}$ is the current flowing through $C_{st}$. $R_m$ is the current measuring resistor, $V_2$ denotes the voltage drop across $Z_{s}$ and $C_{st}$, while $V_1$ denotes the voltage drop across $R_{m}$ and $C_{m}$. $I_{cm}$ and $I_m$ represent the current flowing through $C_{m}$ and the measured current respectively.

Figure 1 shows the electric circuit of a potentiostat in a two-electrode cell configuration. $Z_{s}$ in Fig. 1 is the impedance of the system being measured while $C_{st}$ is the stray capacitance between the WE–CE/RE. This is the stray capacitance of interest as the effect of the other stray capacitance in the potentiostat (stray capacitance from RE—ground and from RE–CE) becomes negligible when a low impedance RE is used^23^. The measured impedance ($Z_{m}$) can be described as^23^:

1 $$\begin{aligned} Z_{m} = \dfrac{V_{2}}{I_{m}} = \dfrac{V_{2}}{V_{1}} R_{m} \end{aligned}$$

where $V_{2}$ is the voltage drop across $Z_{s}$ and $C_{st}$ while $I_m$ is the measured current. $V_{2}$ can be described as:

2 $$\begin{aligned} V_{2} = I_{s} Z_{s} Z_{V_{2}} = \dfrac{I_{st}}{j \omega C_{st}}Z_{V_{2}} \end{aligned}$$

where $I_{s}$ and $I_{st}$ are the current flowing through $Z_{s}$ and $C_{st}$ respectively. $Z_{V_{2}}$ is the transimpedance of the electrometer ($V_2$) which is unknown. $V_{1}$, which measures the current as a voltage drop across $R_m$ is given by:

3 $$\begin{aligned} V_{1} = I_{m} Z_{V_{1}} R_m = I_{Cm} \dfrac{1}{j \omega C_m} Z_{V_{1}} \end{aligned}$$

where $Z_{V_{1}}$ is the impedance of $V_1$ and $I_{Cm}$ is the current flowing through $C_m$. Equation (1) can then be rewritten as:

4 $$\begin{aligned} Z_{m} = \dfrac{I_{s} Z_{s} Z_{V_{2}} }{I_{m} Z_{V_{1}}} \end{aligned}$$

The total current ($I_{T}$) is given by $I_{T} = I_{s} + I_{st} = I_{m} + I_{Cm}$, subsequently $I_{T} = I_{m}(1 + j \omega C_m R_m)$ and $I_{m} = I_{T}/ (1 + j \omega C_m R_m)$. Defining $Z_{V_{1}}^\prime = Z_{V_{1}} / (1 + j \omega C_m R_m)$, Eq. (4) can be rewritten as:

5 $$\begin{aligned} Z_{m} = \dfrac{I_{s} Z_{s} Z_{V_{2}} }{I_{T} {Z_{V_{1}}^\prime }} \end{aligned}$$

6 $$\begin{aligned} Z_{m}= & {} \dfrac{I_{s} Z_{s} Z_{V_{2}} }{(I_{st} + I_{s}){Z_{V_{1}}^\prime }} \end{aligned}$$

7 $$\begin{aligned} \dfrac{1}{Z_{m}} = \dfrac{Z_{V_{1}}^\prime }{Z_{V_{2}} Z_{s}} + \dfrac{ I_{st} {Z_{V_{1}}^\prime }}{ I_{s} Z_{s} Z_{V_{2}}} \end{aligned}$$

8 $$\begin{aligned} \dfrac{Z_s}{Z_{m}}= & {} \dfrac{Z_{V_{1}}^\prime }{Z_{V_{2}}} + \dfrac{ I_{st} {Z_{V_{1}}^\prime }}{ I_{s}Z_{V_{2}}} \end{aligned}$$

Redefining $I_s$ and $I_{st}$ from Eq. (2) will lead to $I_s = V_2 / Z_{V_{2}} Z_s$ and $I_{st} = V_2 j \omega C_{st}/ Z_{V_2}$. Substituting the term $I_{st}/I_{s}$ in Eq. (8) with $j \omega C_{st} Z_s$ leads to:

9 $$\begin{aligned} \dfrac{Z_s}{Z_{m}} = \dfrac{Z_{V_{1}}^\prime }{Z_{V_{2}}} + \dfrac{j \omega C_{st} Z_s {Z_{V_{1}}^\prime }}{Z_{V_{2}}} \end{aligned}$$

The transimpedance of the potentiostat $Z_{tr}$ can be defined as $Z_{tr} = Z_{V_{1}}^\prime /Z_{V_{2}}$. Subsequently, Eq. (9) results to:

10 $$\begin{aligned} \dfrac{Z_s}{Z_{m}} = Z_{tr} (1 + j \omega C_{st} Z_s ) \end{aligned}$$

Equation (10) indicates a linear relationship between the relative admittance of the system being measured (${Z_{s}}/{Z_{m}}$) and ${Z_{s}}$. $Z_{tr}$ is frequency-dependent and extrapolated at each frequency as explained in S2 of the supporting information, while $C_{st}$ reported in this paper is an average of the $C_{st}$ at different current ranges.

## Results and discussions

### Extracting the transimpedance and stray capacitance

To extract the transimpedance of the potentiostat ($Z_{tr}$) and stray capacitance ($C_{st}$), the impedance of high precision resistors at different current ranges of the potentiostat from ten times to one-tenth of the corresponding $R_m$ of the current range was measured. $Z_{tr}$ and $C_{st}$ were extracted from the intercept and slope of the first-order polynomial fit of the relative admittance (${Z_{s}}/{Z_{m}}$) versus ${Z_{s}}$ according to Eq. (10). The stray capacitance due to the potentiostat and cell setup $C_{st}$ is obtained from the slope of the plot of the high-frequency data point of $j\omega C_{st}$ versus $\omega$ (Fig. S2 in the supporting information). The stray capacitance ($C_{st}$) was estimated to be 240 ± 5 pF.

### Bandwidth of the potentiostat

**Figure 2:**
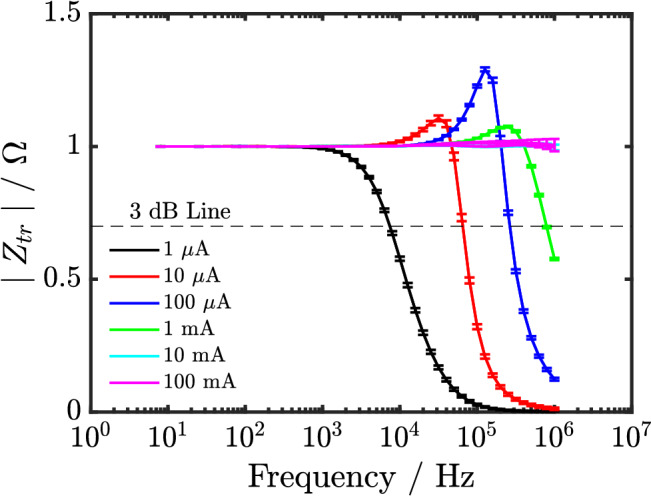
The plot of the magnitude of the transimpedance of the potentiostat ($\mid Z_{tr} \mid$) at different current range versus frequency. The plot shows the 3 dB line, which allows for the bandwidth of the potentiostat at different current ranges to be read off.

The bandwidth of the potentiostat in terms of the transimpedance ($Z_{tr}$) at different current ranges is shown in Fig. 2. Ideally, $\mid Z_{tr} \mid$ should be unitary at all frequencies. However, the result shows that the bandwidth (frequency where the modulus of $Z_{tr}$ deviates from the ideal unitary value of − 30%), decreases from 800 kHz to 8 kHz when current range decreases from 1 mA to 1 $\mu$A as expected.

### Extracting impedance of the system from measured impedance

The impedance of the various electrochemical systems (resistors at different current ranges, $[\hbox {Fe(CN)}_6]^{3-/4-}$ redox couple and nickel hexacyanoferrate) devoid of the instrumental artefacts were extracted from the measured impedance using Eq. (11) and the estimated $C_{st}$ of the potentiostat and $Z_{tr}$ of the current range of interest.

11 $$\begin{aligned} {Z_{corr}} = \dfrac{Z_{tr} Z_m}{1 - j \omega C_{st} Z_m Z_{tr} } \end{aligned}$$

### Resistor

**Figure 3:**
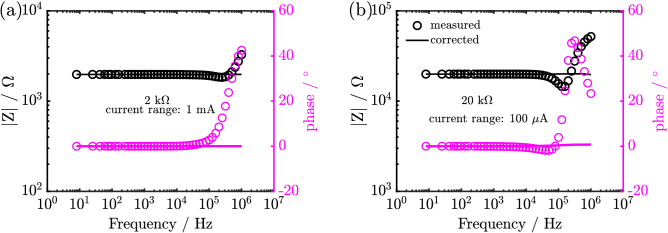
Correction method applied to 2 k$\Omega$ at 1 mA and 20 k$\Omega$ at 100 $\mu$A current range. *circle* represents the measured impedance, while *dash line* represents the corrected impedance using the proposed method.

The use of Eq. (11) to obtain the real impedance from the measured impedance of various resistors is illustrated in this section of the paper. The magnitude and phase of the impedance of 2 k$\Omega$ at 1 mA and 20 k$\Omega$ at 100 $\mu$A current range is shown in Fig. 3.

Ideally, the magnitude of the impedance of a resistor is constant and the phase is zero at all frequencies. The measured impedance (circle in Fig. 3) was observed to deviate from its constant magnitude and zero phase at ca. 100 kHz in both cases. The deviation can be attributed to instrument artefacts. Using the correction method, the real impedance (unitary magnitude and zero phase at all frequencies) can be extracted (continuous lines in Fig. 3). This illustrates that the proposed correction method can be used for extending digitally the bandwidth of the potentiostat. The limit of the correction method for the different current ranges is shown in section S3 of the supporting information.

### Redox couple

**Figure 4:**
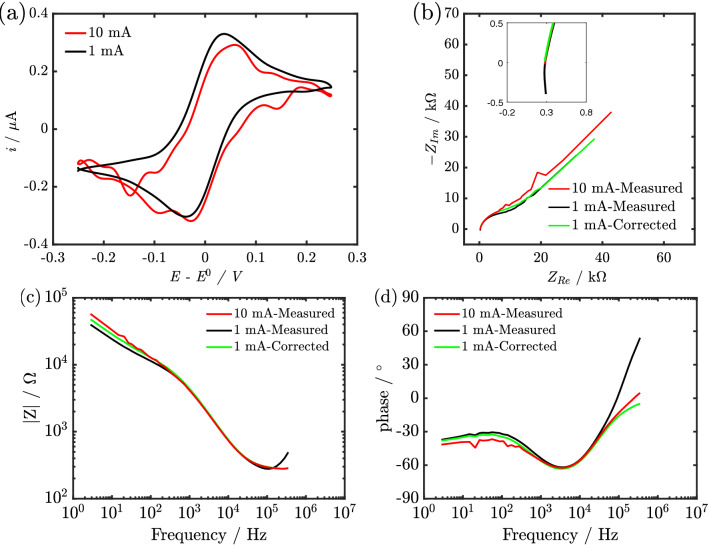
(**a**) Quasi Voltammogram obtained at the different current range for 250 $\mu$m Pt electrode in 5 mM $\hbox {K}_{3}[\hbox {Fe(CN)}_{6}]$ solution with 1 M $\hbox {KCl}$ as supporting electrolyte. (**b**) Nyquist plot of impedance spectra obtained for 250 $\mu$m Pt electrode in 5 mM $\hbox {K}_{3}[\hbox {Fe(CN)}_{6}]$ solution with 1 M $\hbox {KCl}$ as supporting electrolyte using 1 mA and 10 mA current range with an insert showing the high-frequency region. (**c**) and (**d**) Magnitude ($\mid Z \mid$) and Phase of impedance spectra obtained for 250 $\mu$m Pt electrode in 5 mM $\hbox {K}_{3}[\hbox {Fe(CN)}_6]$ solution with 1 M $\hbox {KCl}$ as supporting electrolyte using 1 mA and 10 mA current range.

The effect of instrument artefacts and application of the proposed correction method was also investigated using a $[\hbox {Fe(CN)}_6]^{3-/4-}$ redox couple on a 250 $\mu$m Pt electrode in a three-electrode cell configuration. The dynamic impedance spectra were recorded during quasi-cyclic voltammetry, as explained in^31,32,35^. In this case, as discussed previously, the current range has to be selected to obtain undistorted spectra at high-frequency and simultaneously have good quality dc data (quasi-cyclic voltammetry). The uncompensated cell resistance was 275 $\Omega$, in agreement with the conductivity of the solution and the dimension of the working electrode. For measuring such resistance properly at 1 MHz, a current range of 10 mA ought to be used. However, as seen in Fig. 4a, the use of 10 mA current range would strongly reduce the quality of the extracted voltammogram. It is important to highlight that the reduced data quality of the voltammogram at 10 mA does not originate from interactions between the ac and dc signal as shown in section S4 of the supporting information but from the precision of the current amplifier.

Repeating the same experiment with a 1 mA current range improves noticeably the data quality. However, it introduces a distortion connected to the transimpedance of the potentiostat in the form of an inductive component, as observable in the inset in Fig. 4b and the magnitude and phase of the impedance shown in Fig. 4c,d respectively. It is assumed that the potentiostat is at virtual ground and the current is then measured as the voltage drop across $R_m$. However, this is not the case as the frequency increases, rather the characteristic of the current amplifier at high frequencies resembles that of an inductor, hence the potentiostat, in reality, controls the current via an RLC element^6^. The inductive component in the measured impedance, which is an artefact from the current amplifier due to the usage of an inappropriate current range (1 mA), can be compensated by $Z_{V_{1}}$, which is one of the components of $Z_{tr}$. Using the proposed correction method (Eq. (11)) and the extracted $Z_{tr}$ for 1 mA current range, the real impedance can be extracted from the measured impedance as shown in the inset of Fig. 4b (green curve), and Fig. 4c,d. The result indicates that the proposed correction method allows choosing an appropriate current range, without compromising the high-frequency data in the impedance spectra, thus enabling the measurement of dynamic impedance at high-frequency. This is particularly important in the analysis of adsorption phenomena and the study of the double layer at microelectrodes.

### Nickel hexacyanoferrate nanoparticles

In this section, we illustrate furthermore the effect of instrumental artefacts on the data evaluation of more complex systems. The system of choice is nickel hexacyanoferrate (NiHCF) nanoparticles, which have been reported as a promising cathode material for aqueous and non-aqueous battery systems^40,41^ and lithium recovery^41–48^.

**Figure 5:**
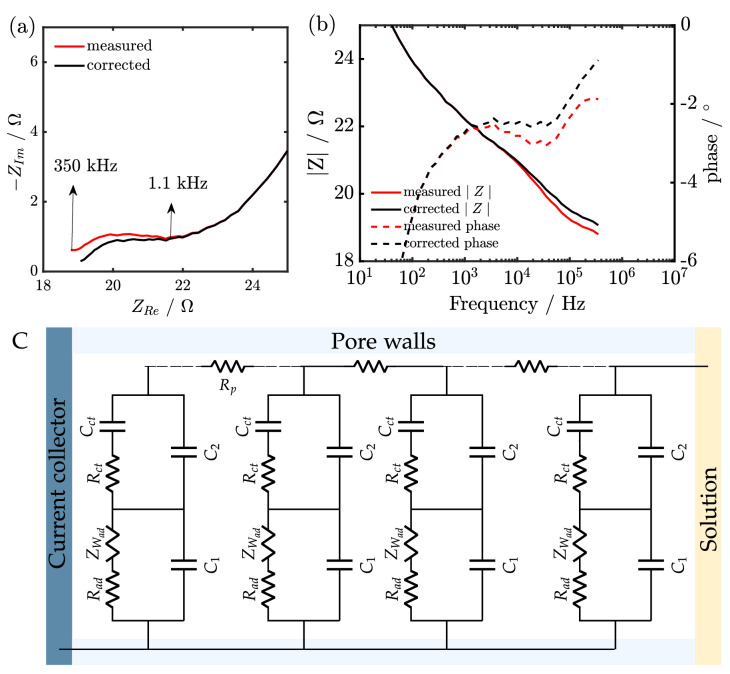
(**a**) and (**b**) Nyquist plot and Bode plot of NiHCF nanoparticles in 0.5 M $\hbox {K}_{2}\hbox {SO}_4$ within the frequency range of 350 kHz to 2.8 Hz at 0.50 V during the cathodic scan respectively. (**c**) Equivalent circuit of the porous electrode including the equivalent circuit obtained from modelling the reversible insertion as two step process treating the capacitance of adsorption ($C_{ad}$ ) as a short circuit and describing the mass transport in the solid with a capacitor^17^.

For intercalation materials such as NiHCF nanoparticles, the impedance is normally acquired over a wide range of frequencies as it allows for the study of the various physicochemical processes occurring during the intercalation process such as (de)adsorption, interfacial charge transfer and mass transport in the solid. The Nyquist plot of the impedance spectra in Fig. 5a shows two RC time constants followed by a straight line attributed to mass transport in the solid. A comparison of the high-frequency data points between the measured and corrected impedance indicates that the former is shifted with respect to the latter (Fig. 5a,b), which can be attributed to the transimpedance of the potentiostat. Although the measured impedance can still be fitted using the same equivalent circuit (Fig. 5c)^17^, the extracted kinetic parameters are different, as shown in Table 1. A 5.6 % error was observed for the extracted capacitance of the double layer ($C_{dl}$). The error introduced by instrumental artefacts exceeds 10 % for other parameters in the high-frequency region such as adsorption resistance ($R_{ad}$), charge transfer resistance ($R_{ct}$) and the resistance of the pores ($R_{p}$). As expected, kinetic parameters of physicochemical processes occurring in the low frequency were unaffected.

**Table 1:** Extracted parameters from fitting the measured and corrected impedance of NiHCF nanoparticles in 0.5 M $\hbox {K}_{2}\hbox {SO}_4$ with the equivalent circuit (Fig. 5c. $R_{u}$ denotes the uncompensated cell resistance, $C_{dl}$ is the capacitance of the double layer, $R_{ad}$ is the adsorption resistance, $\sigma _{ad}$ is the Warburg coefficient of the cation in the liquid, $R_{ct}$ is the charge transfer resistance, $C_{ct}$ is the capacitor describing the mass transport in the solid and $R_{p}$ is the pore resistance^49^.

Parameters	Measured impedance	Corrected impedance
$R_{u}$ ($\Omega$)	18.24	18.67
$C_{dl}$ (*F*)	5.64 $\cdot$ 10$^{-6}$	5.98 $\cdot$ 10$^{-6}$
$R_{ad}$ ($\Omega$)	1.39	1.64
$\sigma _{ad}$ ($\Omega \cdot s^{0.5}$)	57.24	57.92
$R_{ct}$ ($\Omega$)	0.86	0.72
$C_{ct}$ (*F*)	6.54 $\cdot$ 10$^{-3}$	6.56 $\cdot$ 10$^{-3}$
$R_{p}$ ($\Omega$)	4.44	5.56

### Conclusion

The results shown in this paper indicate that the artefacts arising from transimpedance of the potentiostat and stray capacitance between WE–CE can be properly corrected by taking into account the equivalent circuit of the potentiostat and the frequency response of the voltage and current amplifiers. This is particularly important in dynamic impedance spectroscopy when a broadband signal is used to measure the impedance in a large range of frequencies. When current ranges above 1 mA are used, the impedance can be acquired with an error less than 1% in magnitude and 1$^\circ$ in phase. The bandwidth of the potentiostat decreases with decreasing current range. The possibility to estimate the correct impedance from the measured impedance was illustrated using 2 k$\Omega$ and 20 k$\Omega$ resistors. The results indicate that it is possible to acquire the proper impedance values of the resistors up to 1 MHz despite the use of current ranges with bandwidth as low as 100 kHz, thus extending digitally the bandwidth of the potentiostat. Using the proposed correction method, the high-frequency artefacts (inductive behaviour) observed in the measured impedance of a redox couple acquired using a current range of 1 mA were properly corrected. This highlights that the proposed correction method allows choosing properly the current range not only for obtaining reliable high-frequency impedance data, but also for measuring appropriately the cyclic voltammetry. The need to correct measured impedance data was illustrated using NiHCF nanoparticles, where the quantification of kinetic parameters can be severely compromised by the presence of the high-frequency artefacts.

## Methods

The stray capacitance CE–RE and the transimpedance of the potentiostat were measured using high precision resistors polarized at 0 V in a two-electrode cell configuration. A multisine wave consisting of 45 frequencies covering 5 decades was used as the perturbation signal. The base frequency of the multisine (f$_{\rm{ac}}$) was 1 Hz corresponding to measuring the impedance from 1 MHz to 8 Hz. A summary of the parameters used for the acquisition of the impedance spectra can be found in Table 2.

NiHCF nanoparticles were synthesized using the co-precipitation method reported in^42^. The procedure consists of the simultaneous addition of 120 ml of 50 mM of $\hbox {K}_{3}\hbox {Fe(CN)}_{6}$ and 120 ml of 100 mM of ${\hbox {Ni}(\hbox {NO}_{3})}_{2}$ using a flow-rate of 1 ml per minute to 60 ml of distilled water held at $70^\circ$C in a water bath while stirring constantly^42^. The resulting suspension was sonicated for 30 minutes at $70^\circ$C and left overnight to settle. The precipitates were washed with distilled water and dried for 12 hours at $60^\circ$C . To prepare NiHCF electrodes, 3 mm glassy carbon electrodes (GCE) were polished using consecutively a 2000 grit sandpaper rotating at 400 rpm, diamond suspension (Struers) of 0.250 $\mu$m and 0.1 $\mu$m rotating at 300 rpm. The polished electrodes were rinsed with distilled water and sonicated for three minutes to remove any diamond suspension left on the electrode surface. The cleaned GCE surface was coated using the doctor blade method (200 $\mu$m) with NiHCF slurries made with a weight ratio of 80:10:10 of the synthesized NiHCF, carbon C65 (Timcal, Bodio Switzerland) and polyvinylidene fluoride (PVDF) solution in NMP (Solef S5130, Solvay) respectively.

The electrochemical measurements of the NiHCF nanoparticles and the redox couple were carried out in a three-electrode cell setup in coaxial cell geometry to reduce artefacts arising from electrode configuration^4^. The electrolytes used for NiHCF nanoparticles and redox couple measurement were 0.5 M $\hbox {K}_{2}\hbox {SO}_{4}$ and 20 mM $\hbox {K}_{3}[\hbox {Fe(CN)}_{6}]$ in 1 M $\hbox {KCl}$ respectively. The working electrode (WE) was the 3 mm modified NiHCF nanoparticle electrode or a 250 $\mu$m platinum electrode depending on the system under investigation, while the counter electrode (CE) was a platinum mesh (Labor Platina). The reference electrode (RE) was a capacitively-coupled low-impedance (20 $\Omega$) $\hbox {Ag/AgCl}$ (3M KCl) fabricated by coiling a 0.1 mm platinum wire around the tip of a $\hbox {Ag/AgCl}$ reference electrode and connecting it through a 100 nF capacitor to the $\hbox {Ag}$ wire^15^. The use of this low impedance reference electrode reduces high-frequency artefacts associated with the cell setup^4,15,18^.

**Figure 6:**
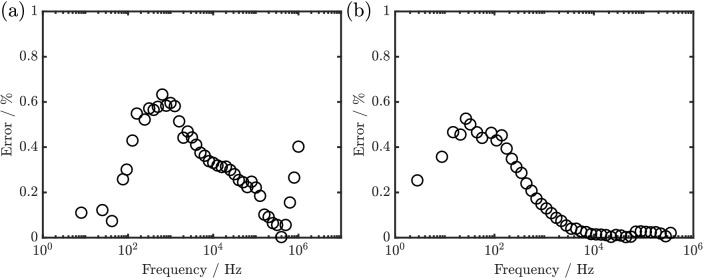
Plot of the estimated error due to the nonlinear component of the fundamental frequencies of dynamic impedance acquired using a multisine intensity of 50 mVpp in (**a**) $[\hbox {Fe(CN)}_{6}]^{3-/4-}$ redox couple (**b**) nickel hexacyanoferrate.

Dynamic impedance spectra were acquired by perturbing the WE electrode held at predetermined potential ($E_h$), with a combined quasi-triangular waveform (QTW) and a multisine wave created by a waveform generator. The frequency of the QTW (f$_{\rm{dc}}$) and the amplitude ($\Delta$U$_{\rm{dc}}$) used for the $[\hbox {Fe(CN)}_{6}]^{3-/4-}$ redox couple and NiHCF nanoparticles are shown in Table 2, which results in an equivalent scan rate of 50 mVs$^{-1}$ and 8 mVs$^{-1}$ respectively. Dynamic impedance spectra were acquired between 1 MHz and 8 Hz for the $[\hbox {Fe(CN)}_{6}]^{3-/4-}$ redox couple and 350 kHz to 2.8 Hz for the NiHCF nanoparticle using the base frequency of the multisine (f$_{\rm{ac}}$) in Table 2. The amplitude of the multisine ($\Delta U_{ac}$) used for both the $[\hbox {Fe(CN)}_{6}]^{3-/4-}$ redox couple and NiHCF nanoparticles was 50 mVpp resulting in negligible error from the nonlinear components as shown in Fig. 6. Details of the effect of the intensity on the multisine can be found in the supporting information. The input (voltage perturbation) and output (current response) signals were measured using a two-channel oscilloscope 4262 PicoScope (Pico technology). The measured impedance was extracted with a home-made MATLAB scripts using one of the heuristic description of dynamic impedance^32,35^:

12 $$\begin{aligned} Z' = \frac{iFT[\Delta U(\omega ).g(\omega '-\omega ),bw]}{iFT[\Delta I(\omega ).g(\omega '-\omega ),bw]} \end{aligned}$$

where $\Delta$ $U$ and $\Delta$ $I$ are the Fourier transform of the potential and current signals respectively, $\omega$ is the angular frequency, *g* is a quadrature filter function, and *bw* is the bandwidth of the quadrature filter (Table 2). More details about the application of the quasi-triangular waveform, dynamic multi-frequency analysis and the data extraction and evaluation can be found in^31,32,35^.

**Table 2:** Summary of DMFA parameters used in the acquisition of dynamic impedance.

Parameters	Resistors	$[\hbox {Fe(CN)}_{6}]^{3-/4-}$	NiHCF
$E_h$ (V)	0	0.25	0.1
f$_{\rm{dc}}$ (mHz)	–	50	5
$\Delta$U$_{\rm{dc}}$ (mVpp)	–	250	400
f$_{\rm{ac}}$ (Hz)	1	1	0.35
$\Delta$U$_{\rm{ac}}$ (mVpp)	$100^{*}$	50	50
No of Msample	20	200	400
sample rate ($\mu$s)	0.2	0.2	1
current range (mA)	$$10^{**}$$	1	100
*bw* (Hz)	2	2	1

*The amplitude of the multisine was scaled with the measured resistors to avoid errors due to current-voltage amplification. 100 mVpp was used for the equivalent resistor ($R_{m}$) of the current range and the amplitude used for other resistors was scaled by the ratio of the resistor to $R_{m}$ of the current range.

**A current range of 1 mA was also used to investigate the effect of selecting an inappropriate current range.

## Supplementary Information

Supplementary Information.

## Data Availability

The datasets generated during and/or analysed during the current study are available from the corresponding author on reasonable request.
