# Supplementary material for: Estimation and correction of instrument artefacts in dynamic impedance spectra
Source: Sci Rep. 2021 Jan 14;11:1362. doi: 10.1038/s41598-020-80468-x (PMC7809267; doi:10.1038/s41598-020-80468-x)
Supplement: Supplementary file 1 — Supplementary Information. [file 41598_2020_80468_MOESM1_ESM.pdf]

Supporting Information
Estimation and Correction of Instrument Artefacts in Dynamic Impedance Spectra

Collins Erinmwingbovo and Fabio La Mantia

Universität Bremen, Energiespeicher- und Energiewandlersysteme, Bibliothekstr. 1, 28359 Bremen, Germany.

Contents

S1 Effect of the Intensity of the Multisine **2**

S2 Fitting **3**

S3 Limit of the Correction Method at Different Current Ranges **5**

S4 Fourier Transform of Current Response **8**

S1 Effect of the Intensity of the Multisine

The role of the nonlinear response on the noise level of the impedance spectra was investigated. The current response contains in addition to the fundamental frequencies, the corresponding harmonics and intermodulations. The dc component is influenced by the first harmonics while the fundamental harmonic is influenced by the second order harmonics [1]. The signal-to-noise ratio relative to the nonlinear component (R_k) of the response of the fundamental frequency can be described as [1]:

$$R_k = \frac{1}{\Delta U_{ac}^2} \frac{C_k}{\mid Z(\omega_k) \mid} \tag{1}$$

$$C_k = \sum_j \frac{\mid A'(\omega_k, \omega_j) \mid}{A_j^2} \tag{2}$$

where ΔU_{ac} denotes the amplitude of the multisine signal, A' is the second order harmonic response and A_j is the intensity of the fundamental harmonics at frequency f_j. ω_k and ω_j are the k-th and j-th frequency of the multisine signal [1]. Equation 1 can be rewritten as:

$$\frac{1}{Z(\omega_k)} = \Delta U_{ac}^2 \frac{R_k}{C_k} \tag{3}$$

Equation 3 allows for R_k to be estimated from the slope of admittance ($1/Z(\omega_k)$) acquired at different intensity versus ΔU_{ac}^2. The error due to the nonlinear component at the intensity used in the acquisition of the impedance can then be described as:

$$Error = \frac{R_k}{Y_0} \Delta U_{ac}^2 \tag{4}$$

where Y_0 is the intercept of the plot $1/Z(\omega_k)$ versus ΔU_{ac}^2. The result obtained for dynamic impedance of the redox couple using a multisine intensity of 50 mVpp and for NiHCF nanoparticles using a multisine intensity of 50 mVpp is shown in Fig. 6 of the main paper. The result indicates that the error introduced by the nonlinear components at the used intensity of the multisine in both cases were less than 1%. We considered a multi-sine intensity of 50 mVpp as a good trade off between signal intensity and error arising from the nonlinear components.

S2 Fitting

The transimpedance of the potentiostat (Z_{tr}) and the stray capacitance (C_{st}) between WE — CE were extracted using the mathematical description of the electrical circuit of the potentiostat:

$$\frac{Z_s}{Z_m} = Z_{tr}(1 + j\omega C_{st} Z_s) \tag{5}$$

where Z_s is the impedance of the system which was obtained from the low frequency data points, while Z_m is the measured impedance. Z_{tr} was extracted at each frequency from the linear regression (Z_s/Z_m) as a function of Z_s as shown in Fig. S1. The transimpedance (Z_{tr}) was given by intercept with the y-axis, while the slope, normalized by the transimpedance, was used to obtain the value of ωC_{st}. The stray capacitance (C_{st}) was extracted from the average value at each current range, as shown in Fig S1f.

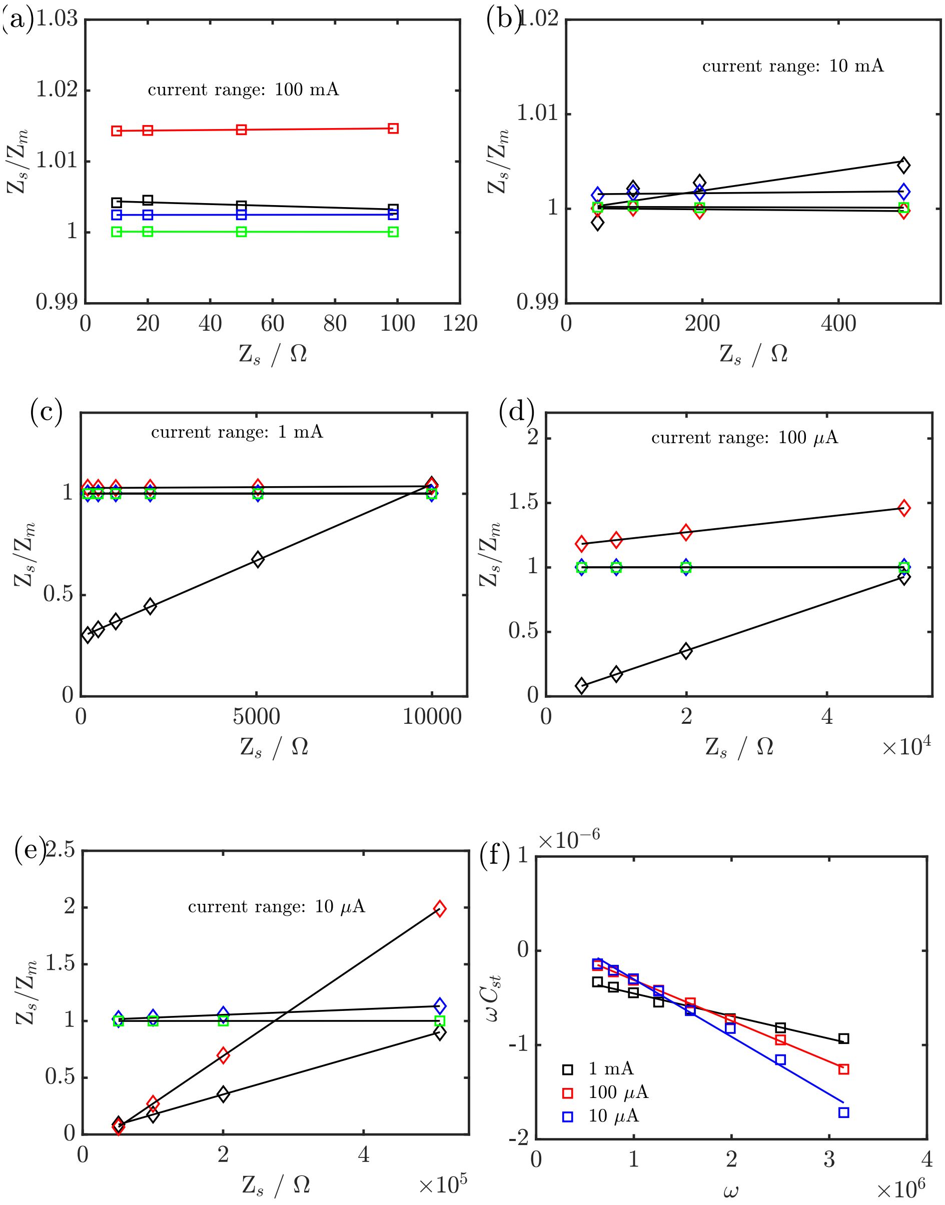

Figure S1: (a) - (e) Polynomial fit of the relative admittance (Z_s/Z_m) versus Z_s of selected frequencies. 993 Hz (green), 10 kHz (blue), 100 kHz (red) and 1 MHz (Black). (f) Polynomial fit of high frequency data points of $j\omega C_{st}$ versus ω.

S3 Limit of the Correction Method at Different Current Ranges

The correction method cannot be used under all conditions, because it tends to amplify all the errors. For this reason, we looked at the limits in the application of the correction. The permissible limit set in this work is an error of 1% for magnitude of the impedance and 1° for phase. The error in magnitude (err $|Z|$) and error in phase (err (φ)) are given by err($|Z|$) = $(|Z_{corr}| - |Z_s|)/|Z_s|$ and err(φ) = $\varphi_{corr} - \varphi_s$, where $|Z|$ and ϕ indicate modulus and phase of the impedance, and the subscript corr and s indicate the corrected and real values respectively.

The results suggest that the error in magnitude and phase were below the limit for all frequencies (up to 1 MHz) for the current ranges from 100 mA to 1 mA. For 100 μA current range (Fig. S3a and Fig. S3b), the result indicates that the error of the measured impedance is below the 1% limit in magnitude and 1° in phase with the exception of 50 kΩ and 100 kΩ, which could be properly measured only up to 100 and 80 kHz respectively. Using 10 μA current range, it was possible to correct the measurement on the 100 kΩ resistance up to a frequency of 200 kHz as shown in Fig. S3c and Fig. S3d. The result for the 10 μA current range show that a resistor of 200 kΩ can be properly measured up to 100 kHz. This limit decreases as the impedance of the resistor increases as shown in Fig. S3c and Fig. S3d.

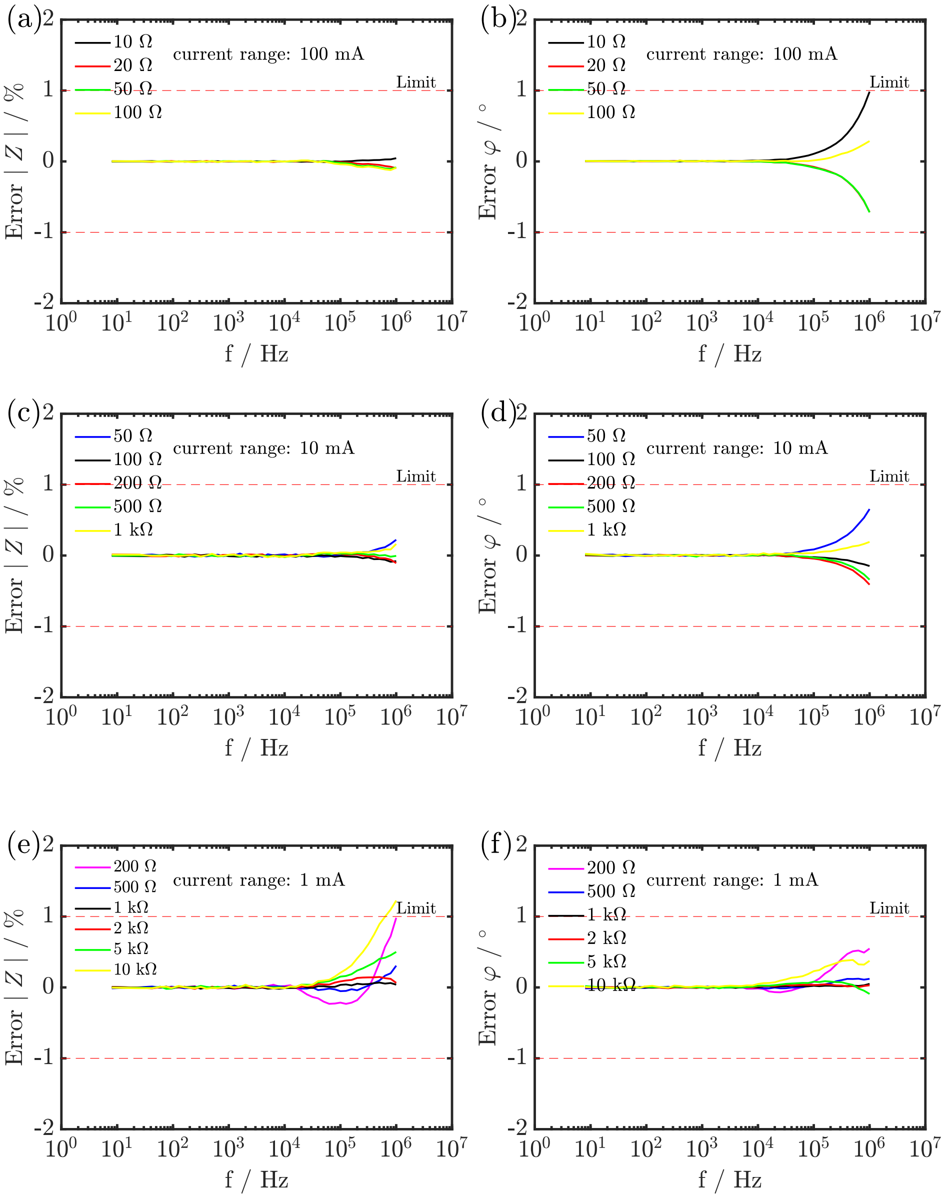

Figure S2: Plot showing the estimated error of the magnitude and phase of the impedance of the resistors at different current range from 100 mA to 1 mA.

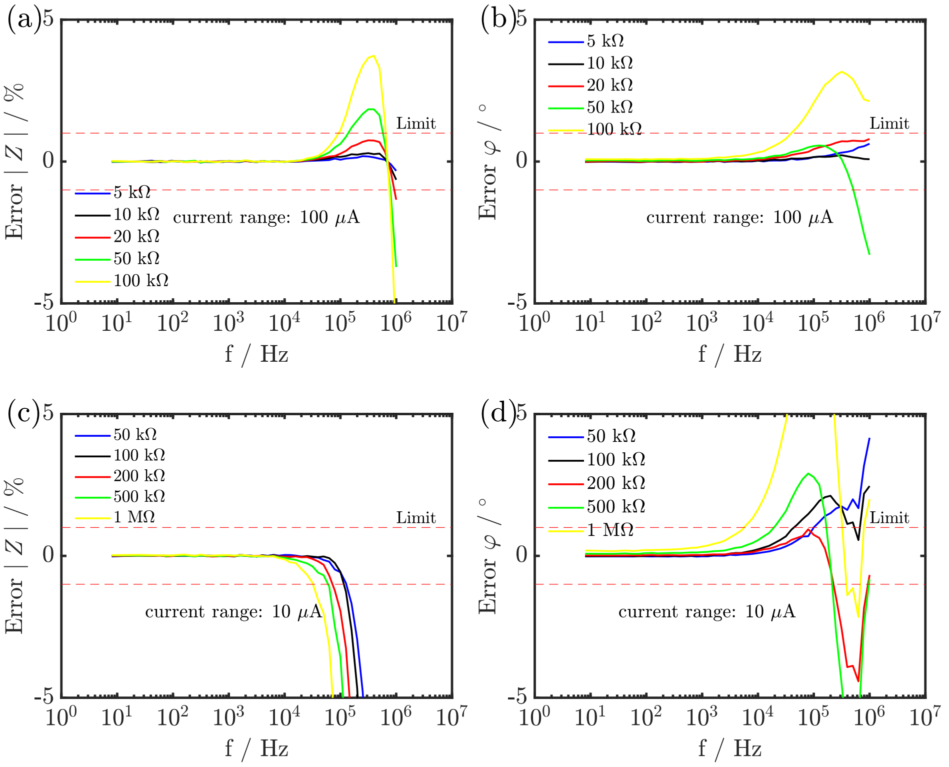

Figure S3: Plot showing the estimated error of the magnitude and phase of the impedance of the resistors at different current range from 100 μA to 10 μA.

S4 Fourier Transform of Current Response

Fig. S4 shows the Fourier transform of the current response $[Fe(CN)_6]^{3-/4-}$ redox couple at different current ranges (10 mA and 1 mA). In both cases, the first frequency in the multisine (8 Hz) does not interact with the frequency of the dc component (50 mHz), indicating that the reduced data quality of the voltammogram in 10 mA does not originate from an interaction of the dc and ac signal.

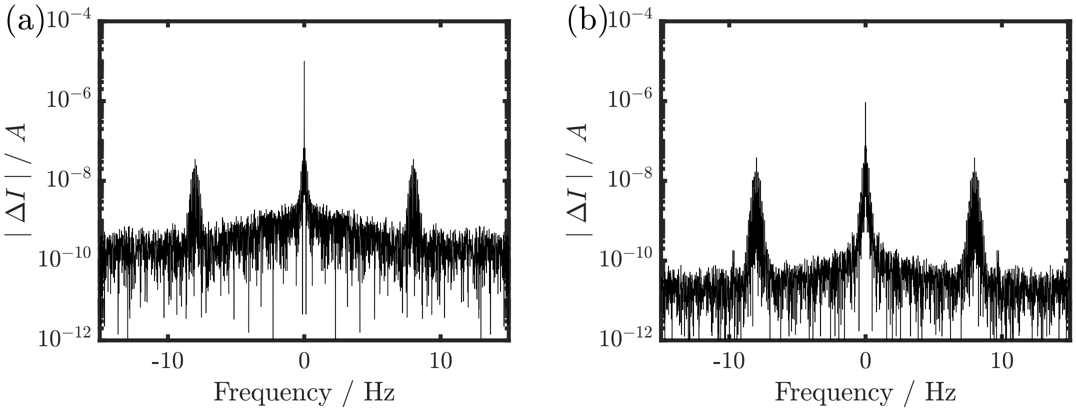

Figure S4: Fourier transform of (a) current response using a current range of 10 mA (b) current response using a current range of 1 mA in $[Fe(CN)_6]^{3-/4-}$ redox couple on a 250 μm Pt electrode.

References

[1] D. Koster, G. Du, A. Battistel, F. La Mantia, Dynamic impedance spectroscopy using dynamic multi-frequency analysis: A theoretical and experimental investigation, Electrochimica Acta 246 (2017) 553–563. doi:10.1016/j.electacta.2017.06.060.
